# A modified regression method to test publication bias in meta-analyses with binary outcomes

**DOI:** 10.1186/1471-2288-14-132

**Published:** 2014-12-17

**Authors:** Zhi-Chao Jin, Cheng Wu, Xiao-Hua Zhou, Jia He

**Affiliations:** Department of Health Statistics, Second Military Medical University, No. 800 Xiangyin Road, Shanghai, 200433 China; Biostatistics Unit, HSR&D Center of Excellence, Veterans Affairs Puget Sound Health Care System, Seattle, Washington 98101 USA; Department of Biostatistics, University of Washington, Seattle, Washington 98195 USA

**Keywords:** Meta-analysis, Observational studies, Publication bias, Smoothed variance, Weighted regression

## Abstract

**Background:**

The tendency towards publication bias is greater for observational studies than for randomized clinical trials. Several statistical methods have been developed to test the publication bias. However, almost all existing methods exhibit rather low power or have inappropriate type I error rates.

**Methods:**

We propose a modified regression method, which used a smoothed variance to estimate the precision of a study, to test for publication bias in meta-analyses of observational studies. A comprehensive simulation study is carried out, and a real-world example is considered.

**Results:**

The simulation results indicate that the performance of tests varies with the number of included studies, level of heterogeneity, event rates, and sample size ratio between two groups. Neither the existing tests nor the newly developed method is particularly powerful in all simulation scenarios. However, our proposed method has a more robust performance across different settings. In the presence of heterogeneity, the arcsine-Thompson test is a suitable alternative, and Peters’ test can be considered as a complementary method when mild or no heterogeneity is present.

**Conclusions:**

Several factors should be taken into consideration when employing asymmetry tests for publication bias. Based on our simulation results, we provide a concise table to show the appropriate use of regression methods to test for publication bias based on our simulation results.

## Background

Meta-analyses of observational studies are as common as the meta-analyses of controlled trials [1]. Combining observational studies is useful in situations where evidence must be synthesized in research areas that are not conducive to randomized controlled trials [2]. However, publication bias (the selective publication of studies based on the magnitude (usually larger) and direction of their findings) presents a particular threat to the validity of meta-analyses [3]. The tendency towards publication bias is greater for observational studies than for randomized clinical trials [4].

More generally, the tendency for smaller studies to show greater effects than larger studies has been termed the “small-study effect” [5, 6]. This effect may be due to publication bias or heterogeneity, which often arises from population differences or methodological diversity across studies. Factors that confuse the relationship between study effect and study size may cause small-study effects [6]. However, to maintain consistency with the previous literature, we consider this distorted relationship to be a form of “publication bias” in this paper.

A convenient way of visualizing the evidence for publication bias is the use of funnel plots [7]. When publication bias is present, the funnel will be asymmetrical, with a tendency for effect sizes to be larger in less precise studies, suggesting a missing “chunk” of the funnel. However, decision-making based on the visualization of funnel plots is rather subjective. Several statistical methods have been developed to examine the publication bias by testing asymmetry in funnel plots. The principle of the existing methods is to test the association between the estimated effect size and the precision of individual studies using rank correlation or regression methods. Rothstein *et al*. provided a detailed discussion of these methods [8]. Two commonly used approaches are Begg’s and Egger’s tests [9, 10]. However, rank correlation-based tests have been criticized for their low power, and most regressions exhibit high type I error rates [5, 11–13]. These tests assume that, under the null hypothesis of no publication bias, there is no association between effect size and precision. This is plausible when the outcome is quantitative, because the assumption of normality implies that the sample mean is statistically independent of the sample variance. This does not hold for binary outcomes [11–16]. Suppose a binary outcome is summarized by the log-odds ratio (logOR). The variance estimators of logOR are statistically dependent of the estimated logOR. Even in the absence of publication bias, as in the simulation study conducted by Rucker *et al*., this dependence induces asymmetry in the funnel plot [15]. The principle behind recently developed methods (such as funnel plot regression [11], Harbord’s score test [14], Peters’ test [12, 17], and Rucker’s arcsine transformed tests [15]) is a reduction in the intrinsic association between the estimated effect size and its estimated asymptotic variance. One potential approach to reduce this association is to use smoothed variance estimates, which have successfully replaced asymptotic variance in simulation studies in the context of random-effects meta-regression. In Berkey’s study, the smoothed estimator of the within-study variance was used in the random effect regression model for meta-analysis to estimate less biased regression coefficients [18].

Sterne *et al*. recommended certain tests for funnel plot asymmetry in meta-analyses with randomized controlled trials [19]. For binary outcomes, Peters’ test, Harbord’s score test, and Rucker’s arcsine-Thompson (AS-Thompson) test were recommended based on simulated meta-analyses with randomized controlled trials. However, observational studies have different characteristics: unbalanced sample sizes in the case and control arms, and possible rare events. Hence, these recommendations are not necessarily appropriate for meta-analyses of observational studies.

In this paper, we develop new regression methods that use a smoothed variance as the precision scale of an individual study to test the asymmetry of funnel plots. In addition, we conduct a comprehensive simulation study based on data from Human Genome Epidemiology (HuGE) to compare the performance of the existing methods with that of the proposed methods. Finally, we make some recommendations based on the simulation results.

## Methods

### Smoothed variance

Suppose we conduct a meta-analysis of *k* studies with binary outcomes. The results for the *i*th study are summarized in Table 1.

**Table 1:** **Notation of outcomes for a single study**

	Case group	Control group	
Exposed	*a*_*i*_	*b*_*i*_	
Unexposed	*c*_*i*_	*d*_*i*_	
			

Using the notation in Table 1, we obtain estimators for logOR and its asymptotic variance as  and , respectively. The estimator of the logOR and its asymptotic variance estimator are intrinsically correlated [14, 15]. To reduce the correlation, we use a smoothed variance to replace the original asymptotic variance or standard error, as in Egger’s regression test.

With *k* studies in a meta-analysis, the estimated smoothed variance for the estimated logOR in the *i*th study is given by


and the standard error is given by .

### Regression models

We introduce two linear regression models based on this smoothed variance. In the first model, we use a weighted regression of *θ*_*i*_ on *se*_*i*_ with weight . We term this method the SVE test (Smoothed Variance regression model based on Egger’s test). The regression model is

*θ*_*i*_ = *α* + *β* × *se*_*i*_ + *ϵ*_*i*_ weighted by , with .

In the second model, which was proposed by Thompson *et al*. [20], we introduce a between-study heterogeneity parameter *τ*^2^. We also regress *θ*_*i*_ on *se*_*i*_, with weight . The regression model is

*θ*_*i*_ = *α* + *β* × *se*_*i*_ + *ϵ*_*i*_ weighted by , with .

The method of moments is used to estimate the between-study variance *τ*^2^ [20, 21]. We term this the SVT test (the Smoothed Variance regression model based on Thompson’s method). The maximum likelihood estimates of *α* and *β* are obtained by least-squares regression with weight *w*_*i*_. The null hypothesis for both methods is *β* = 0, which corresponds to no publication bias.

### Simulation study

To compare the performance of the existing methods with that of the proposed methods, we conducted a comprehensive simulation study. The main simulation procedure is similar to the studies of Peters and Moreno [6, 12, 17]. Our study considers the random effect model. We use Begg’s test [9], Egger’s test [10], Harbord’s score test [14], Peters’ test [12], Schwarzer’s count test [16], the AS-Begg test, AS-Egger test, AS-Thompson test [15], SVE test, and SVT test to examine the publication bias. To make the simulation study more realistic, we reviewed all the Human Genome Epidemiology (HuGE) meta-analyses in the *American Journal of Epidemiology*. We searched the journal using the keywords “Human Genome Epidemiology Review,” “HuGE review,” “polymorphisms,” and “systematic review or meta-analysis”. The information extracted and used in our simulation included the number of individual studies included in each meta-analysis, sample size in the case and control arms, sample size of the included studies, OR values, and heterogeneity. Two investigators performed the literature search and data extraction procedures independently. We now describe the parameters used in the simulation study.

- The number of individual studies included in the meta-analyses were 5, 10, 20, 30, and 60.
- According to the OR values reported in HuGE articles, we set the underlying OR to 1.0, 1.2, 1.4, 1.6, 1.8, and 2.0.
- The average event rates were sampled from the uniform distributions (0.3, 0.7) and (0.1, 0.3), corresponding to the common event rate and rare event rate, respectively, as in previous studies [6, 12, 13, 16, 17].
- Following the simulation reported by Peters *et al*. [12], the between-study variance *τ*^2^ was set to 300%, 100%, 33%, and 1% of the average within-study variance of the fixed effect models. These percentages correspond to *I*^2^ of 75%, 50%, 25%, and 1%, respectively, where the *I*^2^ statistic represents the percentage of the observed between-study variability resulting from heterogeneity rather than chance. As suggested by Higgins [22], we also assigned high, moderate, low, and no heterogeneity to the *I*^2^ values of 75%, 50%, 25%, and 1%, respectively.
- The sample size of the individual studies was generated from the log-normal distribution with a mean of 6.049 and a standard deviation of 0.848, as estimated from the reviewed meta-analyses. The sample size ratios for the combined case and control groups were set to 1:1, 1:2, 1:3, and 1:4. To mimic a real situation, we took the sample ratio for an individual study randomly from a triangle distribution [23], rather than from a uniform distribution. Using the total sample size and group ratio, we generated sample sizes *N*_1_ and *N*_2_ for the case and control groups, respectively.
- Using the parameters generated in steps *a* to *e*, we generated data for the *i*th study as follows [6, 24]:
  Here,  and  are the event probabilities in the case and control groups, respectively. *μ*_*i*_ is the average event rate on the *logit* scale, and *θ* is the logarithmic form of the underlying OR. The other notation is the same as in Table 1.
- No publication bias was induced in this step: all the studies would be published regardless of the significance of their results. Step *f* was repeated until the desired number of studies (5, 10, 20, 30, or 60) was obtained. All studies were included in the meta-analysis, which we defined as the meta-analysis without publication bias. Ten methods were used to test for publication bias, and the process was repeated 1,000 times. The empirical type I error rates of the tests can be estimated from these simulated data under the null hypothesis that there is no publication bias.
- To estimate the power of the tests for detecting publication bias, we introduced publication bias. The probability of publishing a study is determined by the p-value of each study’s primary outcome. Mild and severe publication bias was introduced as follows. Mild bias occurs when the probability of publishing and including an individual study in a meta-analysis was 0.95 when *p* ≤ 0.05, 0.75 when 0.05 < *p* ≤ 0.5, and 0.5 when *p* > 0.5. In addition, 10% of the most extreme effect sizes were censored. Severe bias occurs when the probability of publishing and including a study was 0.95 when *p* ≤ 0.05, 0.5 when 0.05 < *p* ≤ 0.5, and 0.25 when *p* > 0.5. Again, 10% of the most extreme effect sizes were censored. Random numbers were generated from a uniform distribution (0, 1). The probability of inclusion was compared to the generated random number to determine whether each study would be included in a meta-analysis. This was repeated until the desired number of studies (5, 10, 20, 30, or 60) had been obtained. The 10 methods mentioned above were used to test for publication bias and the whole process was repeated 1,000 times. The power of the tests was estimated from these datasets. The Monte Carlo error was around 0.0095.

The power of asymmetry tests largely depends on the number of studies included in a meta-analysis, which is generally small. This limits the power of the test. Therefore, following previous studies [5, 10, 15], we used *p* ≤ 0.10 as evidence for publication bias. The R programming language was used to conduct the simulations [25].

## Results

### Simulation results

In total, 2,880 combinations were simulated (five different numbers of included studies × six different OR values × two average event rates × four levels of heterogeneity × four sample size ratios between the case and control groups × three different levels of publication bias: no bias, mild bias and severe bias). Because of space limitations, we present only the results under the common event rate and the balanced sample size ratio.

**Figure 1:**
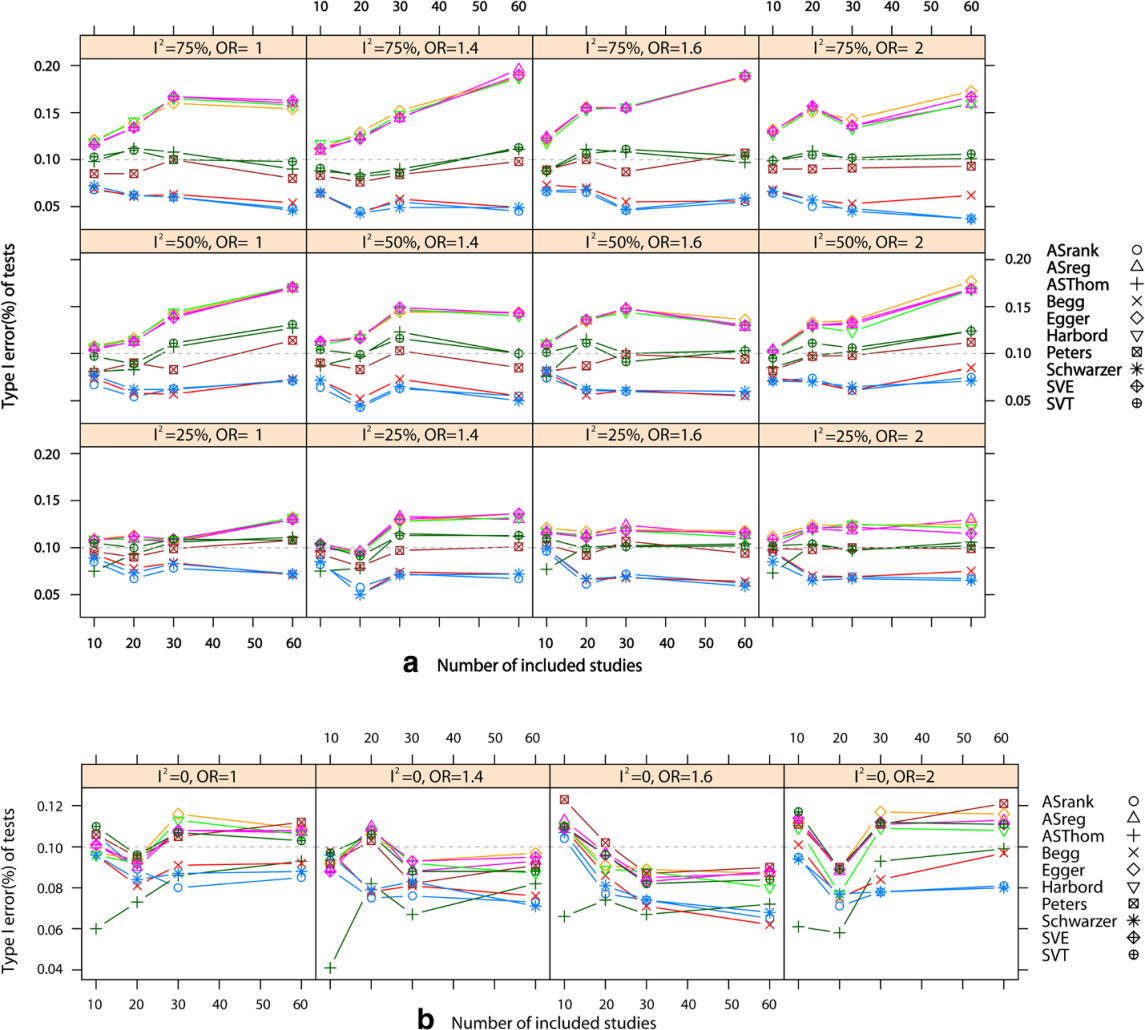
**Empirical type I error rate.** Empirical type I error rate with respect to the number of included studies and different OR values and heterogeneity: ***a***. Type I error rate of 10 tests with heterogeneity; ***b***. Type I error rate of 10 tests without heterogeneity. Nominal significance level is 0.10.

**Figure 2:**
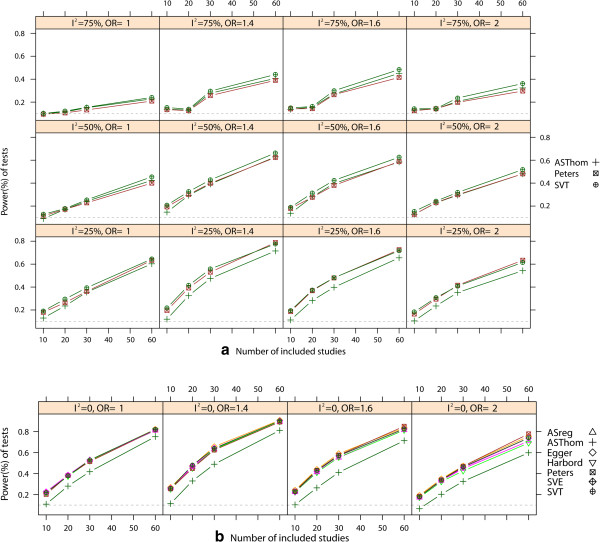
**Statistical power of the tests.** Power with respect to the number of included studies in meta-analyses with severe publication bias and different OR values and heterogeneity: ***a***. Power of three regression tests with heterogeneity; ***b***. Power of seven regression tests without heterogeneity; Nominal significance level is 0.10.

The empirical type I error rates are shown in Figure 1. Only three regression tests (Peters’ test, AS-Thompson test, and our SVT test) had appropriate type I error rates, regardless of the degree of heterogeneity and the number of included studies. In general, the rank correlation tests were more conservative than the regression tests in all scenarios. We observed that the type I error rates of the three rank correlation tests and the other four regression tests diverged from the nominal level as the heterogeneity or number of included studies increased. However, when *I*^2^ was close to zero, all of the regression tests had appropriate type I error rates, except for the AS-Thompson test.We compared the power of the three tests that had appropriate type I error rates in the presence of heterogeneity. From the results shown in Figure 2, we can conclude that our newly developed SVT test is the preferred method. This test exhibits a higher power than Peters’ test and the AS-Thompson test in almost all scenarios. The AS-Thompson test is a suitable alternative when moderate or severe heterogeneity is present. However, the AS-Thompson test is rather conservative when no heterogeneity or mild heterogeneity is present. Peters’ test can be considered a complementary method in such cases. However, several other regression methods can also be used to detect asymmetry in the absence of heterogeneity. The number of individual studies included was another important factor in determining the power of a test. The power of Peters’ test, the AS-Thompson test and the SVT test increased with the number of included studies. All test powers were very low when fewer than 10 studies were included (data not shown). When the event rate and sample size ratio of two groups were considered, Harbord’s test was preferable to the others under the combination of a rare event and larger size ratio between two arms in the absence of heterogeneity. However, the power of our SVT test was only slightly lower than that of Harbord’s test for this combination.

In general, rank correlation tests are more conservative than regression methods. Thus, we do not recommend using rank correlation-based models to test for publication bias. To make these tests more accessible to less technical readers, Table 2 describes which regression method is most appropriate to test for asymmetry in funnel plots based on the results of our simulation study. This table is very easy to read. As the number of included studies increases, the power of the tests also increases, but the relative ranks for the test powers do not change. Therefore, we do not list this factor in the table. This table is applicable to all meta-analyses with more than 10 individual studies. For example, if a meta-analysis with 15 studies has an *I*^2^ value of around 50%, size ratio of approximately 1, and an event rate close to 0.60, we search the table for a method indicated by solid circles with a moderate level of heterogeneity and a size ratio of 1:1, i.e., the SVT test, Peters’ test and the AS-Thompson test. However, the SVT test always has a slightly higher power than Peters’ test and the AS-Thompson test.

**Table 2:** **Recommendation about using the regression methods to test the asymmetry of funnel plot**

Heterogeneity	Size ratio	SVT	SVE	Egger	Harbord	Peters	AS-Egger	AS-Thompson
No	1:1	●	●	●○	●	●	●	–
	1:2	●	●	●	●○	●	●	–
	1:3	●	●	●	●○	●	●	–
	1:4	●	●	●	●○	–	●	–
Low	1:1	●○	–	–	–	●○	–	–
	1:2	●○	–	–	–	●○	–	–
	1:3	●○	–	–	–	●○	–	–
	1:4	●○	–	–	–	●○	–	–
Moderate	1:1	●○	–	–	–	●○	–	●○
	1:2	●○	–	–	–	●○	–	●○
	1:3	●○	–	–	–	●○	–	●○
	1:4	●○	–	–	–	●○	–	●○
High	1:1	●○	–	–	–	–	–	●○
	1:2	●○	–	–	–	–	–	●○
	1:3	●○	–	–	–	–	–	●○
	1:4	●○	–	–	–	–	–	●○

● Applicable for common event.

○ Applicable for rare event.

– Not applicable.

### A real HuGE review example

**Figure 3:**
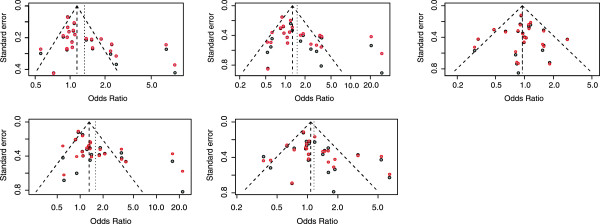
**Funnel plots of a real world example.** Funnel plots of the real HuGE review example under different genetic models. *Black circles*. Standard error estimated from asymptotic variance as the studies’ precision; *Red circles*. Standard error estimated from smoothed variance as the studies’ precision.

We illustrate the use of the tests by detecting the publication bias in a HuGE review that examines the association between ACE-I/D polymorphism and Preeclampsia risk [26]. The meta-analysis of ACE-I/D polymorphism includes 22 studies comprising 2,596 cases and 3,828 controls. The additive model (per-D-allele) reveals a positive association between the ACE-I/D variant and preeclampsia (OR = 1.26, 95% CI, 1.07–1.49). The authors present the results of Egger’s and Peters’ test under the per-allele model. However, these tests may not be appropriate for binary outcomes or severe heterogeneity. Additionally, the authors do not fully describe the publication bias under other genetic models. Table 3 lists all the test results under five genetic models. Given the visualized funnel plots (Figure 3) and resulting *p*-values, we can summarize the results as follows (*α* = 0.10).

**Table 3:** **Resulting** ***p***-**values for testing the publication bias under five genetic models**

Methods	ACE-I/D Polymorphism				
	D ***vs***. I	DD ***vs***. II	ID ***vs***. II	DD ***vs***. ID+II	DD+ID ***vs***. II
	(I ^2^ = 81.1%)*	(I ^2^ = 71.8%)	(I ^2^ = 31.5% )	(I ^2^ = 74.5%)	(I ^2^ = 63.1%)
Begg	0.04	0.11	0.80	0.09	0.17
Schwarzer	0.05	0.19	0.80	0.15	0.27
AS-Begg	0.03	0.03	0.76	0.03	0.23
Egger	0.02	0.02	0.76	0.01	0.09
Harbord	0.02	0.07	0.97	0.02	0.19
Peters	0.004	0.01	0.74	<0.001	0.05
AS-Egger	0.02	0.06	0.97	0.01	0.16
AS-Thompson	0.03	0.13	0.95	0.01	0.18
SVE	0.02	0.06	0.98	0.01	0.15
SVT	0.09	0.21	0.99	0.04	0.24

*I^2^ represents the percentage of between-study variability due to heterogeneity.

1. Comparing Figure 3a and b, we can see that the variance of each individual study became smoother after it was re-estimated, which means that the variance of the studies decreased. The cases of D *vs* I and DD *vs* ID+II in Figure 3b show the asymmetry of the plots.
2. The results of the AS-Thompson test and SVT test agreed in finding publication bias in all comparison groups.
3. In Serrano’s paper, all comparison groups exhibit asymmetry using Egger’s and Peters’ tests, except in the ID *vs*. II group [26]. However, the results of our study suggest that only the per-allele model and the recessive model have such asymmetry.
4. Schwarzer’s count test, a rank-based method, was the most conservative, and did not detect any asymmetry in the four groups. Egger’s test and Peters’ test seemed to give false positive results under the dominant model (DD+ID *vs* II); none of the other eight tests found asymmetry in this group. The positive results given by Egger’s test, Harbord’s test, Peters’ test, the AS-Egger test, AS-Begg test, and SVE test for the DD *vs* II group may have been caused by the inflated type I error rate under severe heterogeneity.

We can conclude that the funnel plots and test results suggest the existence of publication bias under the per-allele model and the recessive model. We have confirmed that the preeclampsia risk associated with the ACE D-allele may largely be a result of publication bias. Note that the publication bias or reporting bias in the HuGE review arises from particular sources. Genetic association studies usually investigate more than one Single Nucleotide Polymorphism (SNP) simultaneously. However, the authors tend to report only SNPs with more favorable results, as well as results under favorable genetic models.

## Discussion

In this research, we have proposed modified regression methods that use smoothed variance estimates to replace the asymptotic variance estimates in Egger’s test when the effect size is the odds ratio. This smoothed variance reduces the correlation between the estimated odds ratio and its variance. Using Thompson’s method, we introduced the parameter *t*^*2*^ of between-study heterogeneity to the regression weight. Our simulation results indicate that the performance of the existing methods and modified methods varies with the number of included studies, levels of heterogeneity, event rates, and sample ratio between the two groups. Neither the existing tests nor the newly developed methods were powerful in all scenarios. However, in most scenarios, our modified regression test, the SVT test, had the most appropriate type I error rate and a relatively high power compared with existing tests.

From the definition of smoothed variance, we can see that the total numbers of cases () and controls () directly influence the smoothed variance of the *i*th study. The exposure rates do not affect this smoothed variance. To some extent, this definition implicitly assumes that the difference in variances between studies is largely a result of differences in the sample size of case and control groups. In fact, Knapp *et al*. found that the order of precision based simply on the sample sizes is exactly the same as that based on the smoothed variance estimates [27]. It is worth noting that all of the methods mentioned above have low power when the sample sizes of the included studies were similar. In this circumstance, methods based on selection models could be used [28, 29].

Compared with the results of Rucker’s simulation study, we found similar conclusions for Peters’ test and the AS-Thompson test [15]. Our results and those of Rucker’s suggest that the AS-Thompson test was more conservative than Peters’ test when there was no heterogeneity and more powerful than Peters’ test when heterogeneity was present. In this latter case, our SVT test became slightly more powerful than Peters’ test as the number of included studies increased. However, their performance is comparable when around 20 studies are included, which is a typical number for many meta-analyses. Therefore, when there are fewer than 20 studies, both tests can be used. As to our test’s apparent superiority over Peters’ test when heterogeneity is present and the number of included studies is larger, we acknowledge that this does not have a solid statistical basis.

From the simulation results, almost all tests performed poorly in the presence of severe heterogeneity. In this case, we recommend exploring possible factors for heterogeneity, rather than testing the asymmetry of funnel plots. The simulation study showed that when few studies (e.g., five) were included in the meta-analyses, the power was very low. As described in the *Cochrane Handbook for Systematic Reviews*, statistical tests for funnel plot asymmetry should only be used when there are at least 10 studies included, because the power of the tests is too low to distinguish chance from real asymmetry when there are fewer studies [30].

Our simulation study has a number of strengths. First, the simulation parameters were mainly extracted from practical reviews, which made the simulation more realistic. Second, we made the sample size between the two arms more realistic for observational studies. The sizes of the two arms in individual studies had a rigorous balance ratio of one in previous simulation studies [12, 14], but this ratio may fluctuate around one or become higher in observational studies. We used the triangle distribution rather than the uniform distribution to randomly generate the sample size ratio between two groups. Taking an arm size ratio of 1:2 as an example, this can be taken from a uniform distribution of (0.30, 0.36). In this situation, the total sample ratio for the case and control groups in a meta-analysis could reach 1:2, but the ratio for each individual study would range from 0.43 to 0.56, which is irrational. The triangle distribution can ensure that the ratio for an individual study comes from a rational range. Additionally, unlike Peters and Rucker, we simulated meta-analyses of rare events, which are not uncommon in HuGE reviews and meta-analyses of adverse events.

Some limitations should be mentioned. Unlike Rucker *et al*. [15, 31, 32], we did not use the Copas selection model to introduce publication bias. We found that the probability of including an individual study in the meta-analysis was very small (nearly 10%) under this model with the previous parameters [15, 31]. This small probability means that the Copas model introduces a very severe publication bias, which is unrealistic [33]. However, the mechanism used to introduce publication bias in our simulation is also somewhat arbitrary. Second, we have not assessed the performance of the rank correlation-based tests when the effect size was under other distributions than the normal distribution. However, in practice, the assumption of normality for the random effect size is rarely verified.

## Conclusions

We have proposed a new version of the regression method with better type I error control and relatively higher power than other methods. We evaluated two newly developed regression methods and other existing methods to test for publication bias under situations that often arise in meta-analyses with observational studies, using the log-odds ratio as the measure of effect size. The purpose of our proposed method is not to replace other tests in all scenarios. Indeed, none of the methods were consistently good at detecting publication bias in all scenarios. Instead, the methods listed in Table 2 are complementary, and should be deployed according to the situation. However, our newly proposed method was generally more robust in most scenarios. Finally, preventing publication bias is better than applying curative methods—the main strategy of prevention is the registration system. The R functions for implementing the proposed method are available from the first author upon request.
